# Optimization, characterization and evaluation of ZnO/polyvinylidene fluoride nanocomposites for orthopedic applications: improved antibacterial ability and promoted osteoblast growth

**DOI:** 10.1080/10717544.2020.1827084

**Published:** 2020-09-30

**Authors:** Yanhai Xi, Wenming Pan, Dan Xi, Xue Liu, Jiangmin Yu, Mintao Xue, Ning Xu, Jiankun Wen, Weiheng Wang, Hailong He, Yanyan Liu, Yue He, Chunjing Guo, Daquan Chen, Xiaojian Ye

**Affiliations:** a Department of Spine Surgery, Changzheng Hospital, Second Military Medical University, Shanghai, China; b Department of Spine Surgery, the Second People’s Hospital of Changshu, Changshu, China; c Shandong Instutute for Product Quality Inspection, Jinan, China; d School of Pharmacy, Yantai University, Yantai, China; e Department of Pharmaceutics, China Pharmaceutical University, Nanjing, China; f State Key Laboratory of Bio-Fibers and Eco-Textiles, Qingdao University, Qingdao, China; g Weifang Industrial Technology Institute of Chinese Medicine, Weifang, China

**Keywords:** Electrospinning, zinc oxide/polyvinylidene, nanoparticles, fluoride composite fiber membrane, piezoelectricity

## Abstract

Herein, electrospun zinc oxide nanoparticle/poly (vinylidene fluoride) (ZnONP/PVDF) composite fiber membranes were designed, fabricated, and tested for improved orthopedic applications. A single factor screening study was conducted to determine the optimal ZnONP/PVDF formulation based on osteoblast (bone forming cells) proliferation and antibacterial properties. Further, ZnONP/PVDF materials were characterized for their morphology, crystallinity, roughness, piezoelectric properties, and chemistry to understand such cell results. The optimal concentration of high molecular weight PVDF (18%, w/v) and a low concentration of ZnONPs (1 mg/ml) were identified for electrospinning at room temperature in order to inhibit bacterial colonization (without resorting to antibiotic use) and promote osteoblast proliferation. Compared to no ZnO/PVDF scaffold without Piezo-excited group,the study showed that on the 1 mg/ml ZnO/PVDF scaffolds with piezo-excitation, the density of SA and *E.coli* decreased by 68% and 56%.The density of osteoblasts doubled within three days(compared to the control). In summary, ZnONP/PVDF composite fiber membranes were formulated by electrospinning showing an exceptional ability to eliminate bacteria colonization while at the same time promote osteoblast functions and, thus, they should be further studied for a wide range of orthopedic applications.

## 1. Introduction

With an increasing aging world population, the treatment of orthopedic problems has become a major challenge. Research in orthopedics not only includes healing of muscles, ligaments or bone, but increasing reducing staphylococcal infections as a result of orthopedic implants (Ribeiro et al., 2012). It is difficult to identify orthopedic materials which can both eliminate bacteria colonization and promote osteoblast functions in the same materials without the use of antibiotics or pharmaceutical agents (which can have a wide range of side effects in the body, such as the development of antibiotic resistant bacteria). In fact, the extensive over use of antibiotics in orthopedics has become such a problem that the Centers for Disease Control in the U.S. have predicted that more people will die from antibiotic-resistant infections than all cancer combined by 2050. To avoid this healthcare epidemic, it is clear that we need new materials which can both improve bone cell functions and inhibit bacteria without resorting to antibiotic use.

So far, there exists no natural material with all these properties, but there is promise. Polyvinylidene fluoride (PVDF) possessed good resistance to chemical corrosion, high temperature, oxidation, radiation and it also has piezoelectric, dielectric, thermoelectric and other special properties making it suitable for orthopedic applications. Moreover, the transformation among various PVDF crystal shapes can be achieved (Agarwal et al., 2008; Noriega et al., 2012) so that the PVDF materials with optimal crystal shapes can be prepared according to implant needs (Kumar & Periman, 1993; Lin et al., 2004). PVDF is considered as a potential bone tissue engineering material because of its piezoelectricity (just like bone) and biocompatibility, however, its applications in the body have been limited in that it has no ability to resist bacteria growth or infection (Rim et al., 2013).

ZnO nanoparticles (ZnONPs) are an ideal choice to reduce bacteria function and resulting infections because ZnO has good antibacterial activity and nanoparticles of Zn promote surface exposure of Zn to further reduce bacteria functions (Jones et al., 2008; Zhang et al., 2010; Azizi et al., 2017). ZnO can decompose free electrons and holes upon exposure to light, which can in turn form reactive oxygen species to kill bacteria. In addition, for the ZnO structure, four equivalent atomic orbitals are formed between Zn and O through sp3 hybridization (Yamamoto, 2001), forming tetrahedral coordination configuration, which provides ZnO with piezoelectric characteristics similar to bone (Lee et al., 2015).

As is well-known, the piezoelectric constant of a polymer is usually lower than inorganic materials (Bar-Cohen & Zhang, 2008) which limits the use of polymers as piezoelectric mimicking orthopedic materials. However, a high piezoelectric simulation can be provided by a composite of PVDF and nanometer ZnO, thus, a ZnO/PVDF composite fiber membrane may possess good biocompatibility (Sultana et al., 2015), piezoelectricity, and antibacterial protpeties suitable for orthopedic applications (Li et al., 2015).

Further, ZnO/PVDF has a wide range of applications. ZnONP/PVDF nanofiber membranes can be formulated into a porous three-dimensional scaffold promoting bone cell infiltration and improved bone growth (Li et al., 2018). The output power of the ZnO/PVDF hybrid structure was found to be enhanced compared to pristine ZnO nanorods and PVDF nanofibers nanogenerators (Fakhri et al., 2019). Maria Kitsara et.al (Kitsara et al., 2019) found that the combination of a highly β-phase electrospun PVDF with oxygen plasma treatment can result in a functional and stable hydrophilic scaffold, which can stimulate excitable cells, like osteoblasts, without the need of an external power source. These findings can lead to new venues in tissue engineering, based on biomimetic 3 D scaffold-electromechanical stimulation fashion. Studies also have shown that the nanocomposite were produced using electrospinning technique in order to have the benefit of piezoelectric properties and non-brittle behavior of ZnO and PVDF for the application in wearable electronic devices (Bafqi et al., 2015).

In this study, PVDF was mixed with ZnONP through electrostatic spinning technology to prepare an ideal composite material with excellent piezoelectric and biocompatibility properties. The resulting ZnONP/PVDF scaffolds were characterized for material properties and their ability to reduce bacteria colonization and at the same time promote osteoblast functions.

## 2. Materials and methods

### 2.1. Materials

PVDF particles, dimethyl formamide and ZnO nanoparticles were purchased from Sigma Aldrich (China). Human osteoblasts were provided by ATCC(China). *Escherichia coli (E.coli)* and *Staphylococcus aureus (SA)* were provided by ATCC(China). Cell proliferation assay kit was provided by Colorimetric (China).

### 2.2. Preparation of composite fiber membrane

PVDF particles (275,000 MW) were dissolved in 9 ml of acetone and 6 ml of dimethyl formamide (DMF). The solution was then stirred at 55 °C for 12 h to be clear and transparent.

15 mg of ZnO nanoparticles (<50 nm) were then added to the solution and sonicated for one hour. The ZnO/PVDF solution was transferred to a 10 ml syringe, mounted on the electrospinning pump syringe (Elite, Beijing Yongkang, China), and samples prepared at a speed of 0.2 mm/min with a 22 g needle under the conditions of 15 kV, a 22 G gun head and a 20 cm receiving distance.

### 2.3. Single factor screening experiments

Four single factor screening experiments were conducted. Four experiments were examined the effect of (1) molecular weight of PVDF;(2) PVDF mass fraction; (3) concentration of ZnO; (4) electrostatic spinning temperature and receiving mode on the content of β crystal.

#### 2.3.1. Effect of molecular weight of PVDF on the content of β crystal

In addition to replacing PVDF with 300,000–400,000 molecular weight, electrospinning samples were prepared again according to the experimental conditions and parameters of above.

The composite fiber membrane prepared above was determined by infrared spectrum, and the content of β crystal in each fiber membrane was calculated.

#### 2.3.2. Effect of PVDF mass fraction on the crystal content of β

The concentration of PVDF may affect the crystallite content.In this experiment, we prepared composite fiber membrane with different PVDF mass fractions.Other experimental parameters are the same.2.1g, 2.7 g and 3.3 g PVDF(275,000) were added in the same preparation method as above, and the rest steps were the same.

#### 2.3.3. Effects of different concentrations of ZnO on the crystal content of β

In this experiment, 0.5, 1, 2, 10 mg/ml ZnONPs and PVDF suspension with mass fraction of 18% (275,000 molecular weight) were prepared for electrostatic spinning under the conditions of 15 kv, 22 G gun head and 20 cm receiving distance (the same method as 2.2.1). The composite fiber membrane prepared was determined by infrared spectrum and the respective crystal content was calculated.

#### 2.3.4. Influence of electrostatic spinning temperature and receiving mode on the content of β crystal

Under the conditions of normal temperature, 70 °C plate heating and 70 °C axis heating, 1 mg/ml ZnONPs and PVDF with mass fraction of 18% (275,000 molecular weight) were investigated in the same way as the preparation of the crystal content of composite fiber membrane prepared by electrostatic spinning at 15 kv, 22 G gun head and 20 cm receiving distance.

### 2.4. IR analysis for β crystal content

β crystals show strong piezoelectricity. Therefore, in electrospinning, the best conditions need to be explored so that PVDF can exhibit maximal piezoelectric properties from the β crystal form. The three main characteristic peaks of the β crystal form appeared in the fiber films at 1431, 1276 and 840 cm^−1^ (Martins et al., 2014; Karan et al., 2015). In order to study the influence of the different factors on the PVDF crystal form, the content of electroactive β crystals in PVDF were calculated as follows (Andrew & Clarke, 2008):

$$F\beta =\left(\frac{A\beta }{\left(\left(K\beta /K\alpha \right)A\alpha +A\beta \right)}\right)$$

A represents the absorbance value of the ɑ crystal at 840,740 cm^−1^, K represents the absorption coefficient of each, and K ɑ and K β are 6.1 × 10^4^ and 7.7 × 10^4^ cm^2^/mol, respectively.

PVDF with different molecular weights (275,000, 300,000–400,000), mass fractions, concentrations of ZnONPs (0.5, 1, 2, 10 mg/ml), electrostatic spinning temperatures and receiving mode on the formation of β crystals was used during the electrospinning system to optimize the following properties important for orthopedic implants.

### 2.5. Characterization of the optimal ZnO/PVDF scaffolds

#### 2.5.1. XRD characterization

In order to determine the optimal PVDF crystallinity, a D8 Advance X-ray Diffractometer (XRD-6100, SHIMADZU, Japan) was used to scan PVDF (solid powder), PVDF spinning, and ZnONP-PVDF (18%, w/v) spinning. The scanning voltage was 40 kV and the current was 40 mA. The scanning range was set to start at an angle of 3.00°, end angle of 80.00°, scanning step 0.02°, and each step time was 0.30 s. Among them, No. 7, 8 and 9 correspond to PVDF (solid powder), PVDF spinning and ZnONP-PVDF (18%, w/v) spinning, respectively.

#### 2.5.2. Morphological analysis by SEM

Scanning electron microscopy (SEM, Hitachi S4800, Japan) was used to investigate the scaffold morphology. Scaffolds were coated with 5 nm of platinum and viewed using an accelerating voltage of 3 kV, current 10 µA and a working distance of ca. 8 mm. Image J software was used to calculate the average fiber diameter of the scaffolds.

#### 2.5.3. FTIR analysis

3 mg of the obtained composite fiber membrane was precisely torn and was mixed with 100 mg of dried KBr after overnight drying. This process lasted at least 30 min. After grinding uniformly, the materials were poured into a tableting device for tableting for 3 min. The pressure was controlled below 10 kPa to prevent chipping due to excessive pressure. After pressing the tablet, it can be put into the FTIR spectroscopy (Perkin Elmer Spectrometer, USA) for testing.

### 2.6. *In vitro* cell studies

#### 2.6.1. Cell culture

Human osteoblasts were used in this experiment. Human osteoblasts were seeded and cultured at 37 °C 5%/95% CO_2_/air in a mixed medium consisting of Osteoblast Basal Medium supplemented with 10% Osteoblast Supplement Mix and 1% Penicillin-Streptomycin.

#### 2.6.2. Antibacterial tests

The ZnONP/PVDF composite fiber membrane was cut into 1 cm^2^ squares and then was flattened on a glass sheet. After sterilizing under UV for 60 min, the samples were placed in a 24-well culture plate. *Escherichia coli(E.coli)* and *Staphylococcus aureus (SA)* at 1 × 10^6^ cells/ml concentration were inoculated at 37 °C in 5%/95% CO_2_/air in 30 g/L Tryptic Soy Broth (TSB) medium in a 24 Non-Tissue Culture Well plate for 6 h. Prior to cell seeding, the plates of piezo-excited groups were transferred into the mechanical stretching machine (MSM) (P1000, WPI, China) for piezoelectric stimulation. The mechanical stretching stimulation was set at a 1 Hz frequency and 10 mm strain. The control group was not treated. After 6 h of culture, the scaffolds and silicone bottom were cut out, put into 3 ml PBS solution and ultra-sonicated (KQ-250, Kunshan, China) for 5 min. After sonication, the eluant solution was diluted 1000 times, and the diluted solution was dropped into an agar plate 3 times to form a colony group. The agar plates were cultured in the incubator for 12 h and taken out for colony counting (*n* = 3).

#### 2.6.3. Osteoblast proliferation tests

The scaffolds were cut into 0.49 cm^2^ squares and attached by a silicone tissue adhesive onto 24-well silicone well plates. The samples and silicone well plates were then sterilized in UV light for 60 min and later rinsed with PBS for 10 min. In the piezoelectric-treatment groups, prior to cell seeding, the well plates with scaffolds attached were transferred into a MSM for piezoelectric stimulation. The mechanical stretching stimulation was set at a 1 Hz frequency and 10 mm strain. The sterilized ZnO/PVDF composite fiber membrane was then placed into the 24-well cell culture plate. Human osteoblasts were seeded at 15,000 cells/cm^2^ in the wells and cultured for 1–3 days. After the incubation, a cell proliferation assay kit (Colorimetric, China) (MTS) was used to assess cell growth.

### 2.7. Statistical analysis

All experiments were conducted a minimum of 4 times with 3 repeats each. All analyzed data are presented as the mean ± SD. The results were analyzed using a Student’s *t*-test with an alpha value of 0.05 (SPSS 25.0) (*p < .05, **p < .01, ***p < .001).

## 3. Results

### 3.1. Preparation optimization and characterization

**Figure 1.**
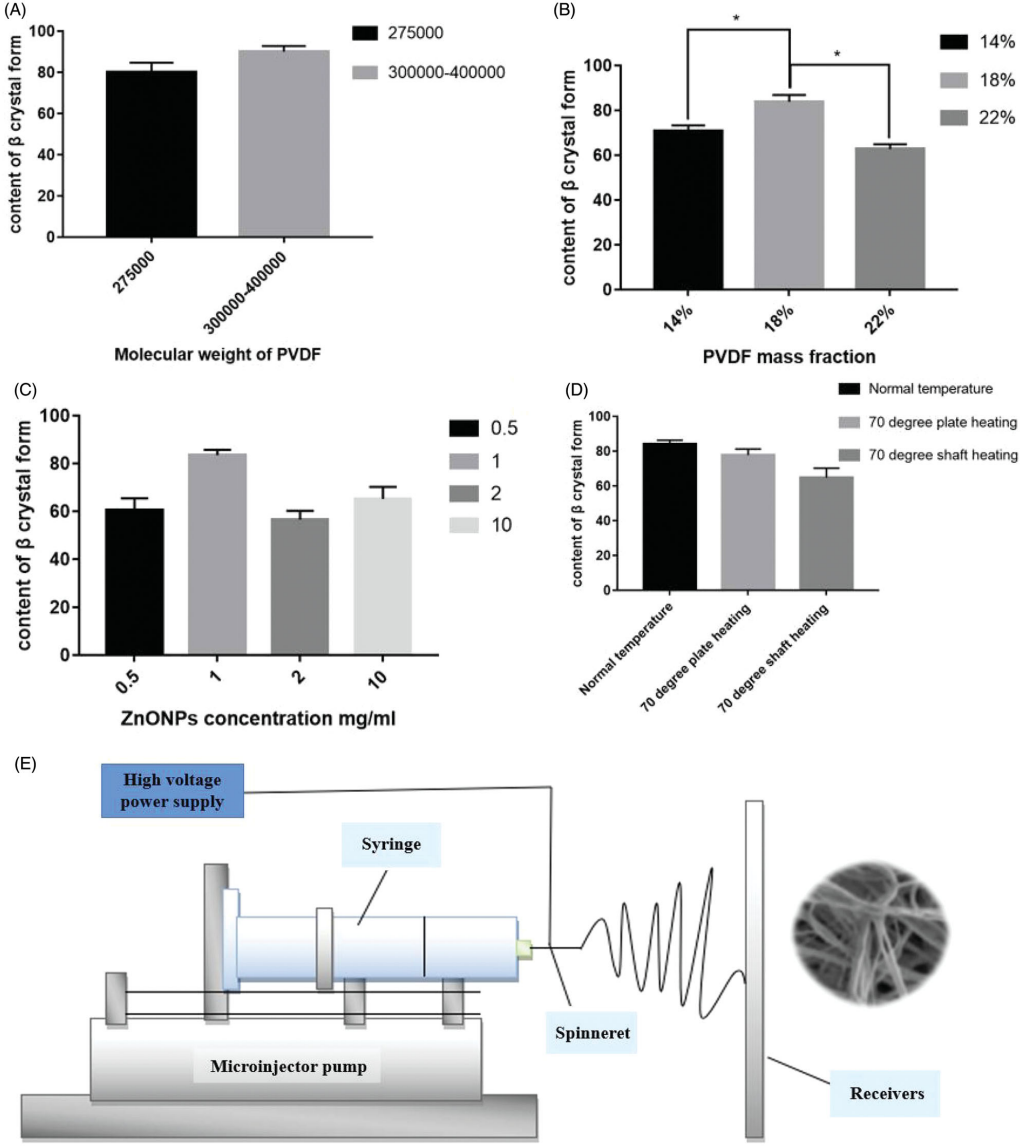
(A) The content of β crystal forms in composite fiber membranes with different molecular weight PVDF. (B) The content of β crystals in the composite fiber membranes with different concentrations of PVDF. (C) The content of β crystals in composite fiber membranes with different concentrations of ZnO. (D) β crystal content of PVDF fiber membrane at different spinning temperatures; Values are mean ± STDEV, *N* = 3, *p < 0.05. (E) The structure of electrospinning.

As shown in Figure 1(A), when the molecular weight of PVDF increased, the relative content of β crystal in the composite fiber membrane increased from 80% to 90%. Because the molecular weight increased from 2.75 × 10^5^ to 3–4 × 10^5^, the PVDF molecules stretched between the chains which are more likely to form the β type crystal. The effect of different concentrations of PVDF on β crystal content is shown in Figure 1(B). The results showed that with the fixed ZnONPs concentration, the relative content of electroactive β crystal formed in PVDF composite fiber membrane increased with PVDF mass fraction increasing from 14% to 22%. The main reason is that the interaction between the PVDF dipole and the surface charge on the surface of ZnONP induced β-phase crystallization, which increased crystal content. In addition, increased PVDF will increase the stretching of its macromolecular chains, making it easier to form β-type crystals (Mao et al., 2014).

The effect of different concentrations of ZnONP on the β crystal form content is shown in Figure 1(C). At a fixed PVDF concentration, as the ZnONPs concentration increased, the relative content of the electroactive β crystal form in the PVDF composite fiber membrane first increased from 60.33%±5.14% to 83.4%±2.28%, and then decreased to 56.53%±3.69%. The main reason for the reduction may be due to formulation of larger particles or particle aggolmerations when higher concentrations of ZnONP were added. Therefore, the nucleation of PVDF was affected, resulting in reduced nucleation, which reduced the crystallinity of the composite fiber membrane (Wu et al., 2012; Aepuru & Panda, 2014).

Figure 1(D) showed the influence of the electrostatic spinning temperature and receiving mode on β crystal form content. It showed that the most β crystals formed on the fiber membrane at room temperature, but under higher temperatures, the β content of the electroactivity decreased to 64.77%±5.41%. The reason may be due to that the solvent evaporated faster and the amount of fibers decreased when heating, which may result in a relative decrease in the beta crystal form.

### 3.2. XRD and SME characterization

**Figure 2.**
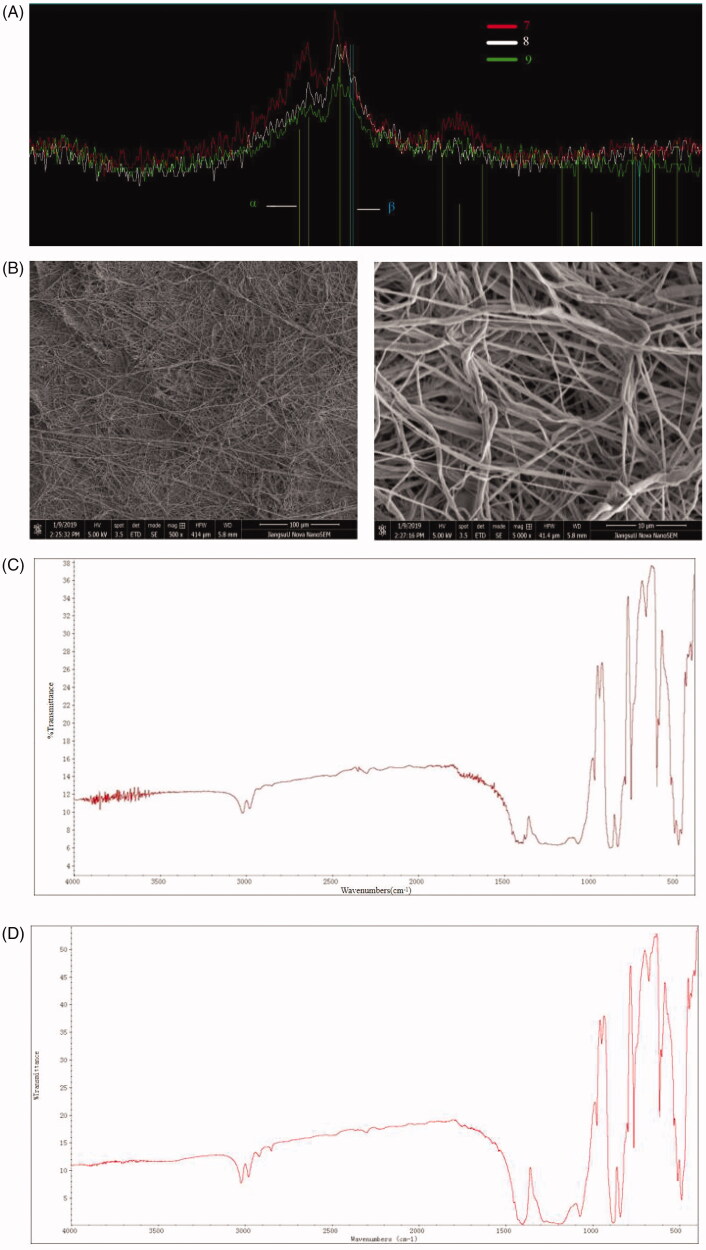
(A) XRD patterns of the three prescriptions, No.7, 8 and 9 correspond to PVDF (solid powder), PVDF spinning and ZnO-PVDF (18%) spinning. (B) Scanning electron microscopy of pure PVDF (left) and PVDF/ZnO (right). (C) Infrared spectrum of PVDF. (D) Infrared spectrum of PVDF fiber membrane.

The Figure 2(A) shows that the crystallinity (Xc) of PVDF (300,000–400,000 MW) after electrospinning increased compared with the PVDF (300,000–400,000 MW) powder. For the scanning electron microscope images of pure PVDF, the fiber surface was relatively smooth and after adding the ZnO nanoparticles, the fiber surface showed slight particle bulges, as shown in the Figure 2(B).

### 3.3. FTIR analysis

Figure 2(C,D) show the infrared spectrum of the PVDF and ZnONP/18%PVDF (300,000–400,000 molecular weight) fiber membrane, with the highest content of β crystals as calculated as 88.42%.

### 3.4. Bacteria density

**Figure 3.**
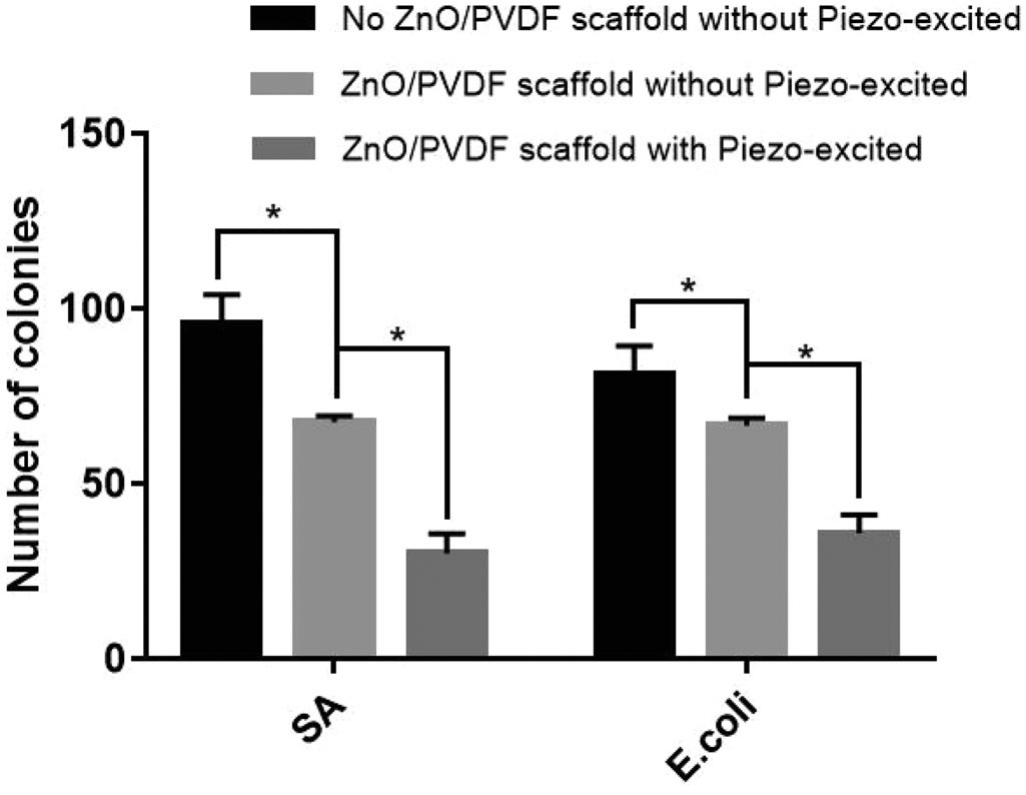
Antibacterial experiment of composite fiber membrane; (Control group (−): No ZnO/PVDF scaffold without piezo-excited, Control group (+): 1 mg/ml ZnO/PVDF scaffold without piezo-excited). Values are mean ± STDEV; *N* = 3, *p < 0.05.

The bacteriostatic results of ZnO/PVDF composite fibers on *E.coli* and *SA* bacteria are shown in Figure 3. Compared to no ZnO/PVDF scaffold without Piezo-excited group, ZnO/PVDF scaffold without Piezo-excited group inhibited bacterial growth, reducing *SA* and *E. coli* density by 20%, 30%, respectively, and ZnO/PVDF scaffold with Piezo-excited group inhibited bacterial growth, reducing *SA* and *E. coli* density by 68%, 56%, respectively. It is noted that ZnO NPs can provide antibacterial properties to scaffolds, even without piezoelectric treatment.

### 3.5. Osteoblast proliferation tests

**Figure 4.**
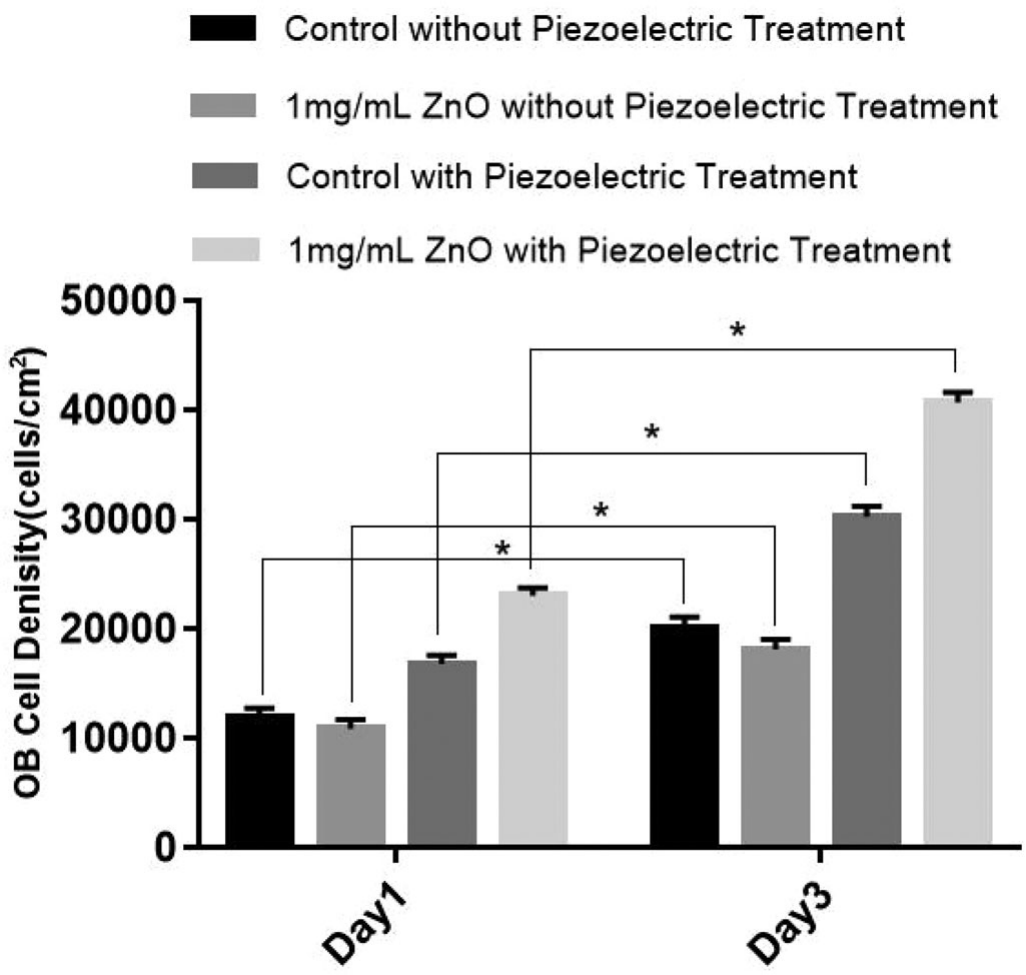
The effect of piezoelectric treatment on osteoblast (OB) density for 1–3 days when cultured on ZnO/PVDF scaffolds (Control group: OB cell Density of Day1); Values are mean ± STDEV *N* = 6, *p < 0.05.

Figure 4 indicates that the osteoblast density on all groups increased from day 1 to day 3. Compared with the control group, the 1 mg/mL ZnO/PVDF NPs without piezoelectric treatment did not significantly promote osteoblast proliferation. With piezoelectric treatment, 1 mg/mL of ZnO/PVDF scaffolds significantly improved osteoblast cell density after day 1 and day 3. The density of osteoblasts doubled within 3 days (compared to the control). Bone is a piezoelectric material that exhibits electricity (Halperin et al., 2004), which can stimulate various biochemical reactions, and energy conversion can be achieved in the process during these biochemical reactions (Tang et al., 2017). Electricity in biosystems (bio-electricity) also promotes growth factor activity and the formation of an extracellular matrix (ECM), which can induce bone reconstruction (Sundelacruz et al., 2013). It was proven that ultrasound-driven piezoelectric stimulation was able to induce calcium influx in neurons, which mediated the enhancement of neurite outgrowth and neural differentiation (Marino et al., 2015). A previous study observed that osteoblasts grown on electrospun PVDF scaffolds showed intracellular calcium concentration transients, suggest that piezoelectric PVDF scaffolds are able to generate a local electric field that activates the osteoblasts (Kitsara et al., 2019). The results indicated that osteoblast proliferation was stimulated by the piezoelectricity generated by the ZnO/PVDF scaffolds.

## 4. Discussion

For treatment of orthopedic problems, the ZnO/poly (vinylidene fluoride (ZnO/PVDF) composite fiber film was prepared by the electrostatic spinning method. Through a series of related single factor optimization, it was found that the higher molecular weight (300,000–400,000) and mass fraction of 18% of PVDF had a great influence on the increasement of β crystal content in ZnO/PVDF composite fiber. In addition, the addition of ZnONPs could not only improve the antibacterial property of the composite, but also increase the content of β crystal in the product PVDF, thus improving the piezoelectric property of the composite. The 1 mg/ml ZnONPs lead β crystal to reach 83.7% as the optimal concentration.

Adding ZnO NPs into the electrospun solution increased the conductivity and charge density of the scaffolds. Increased charge density of the polymer solution could cause a greater repulsion and a greater bending instability during electrospinning, which caused stretching of the fibers,resulting in a smaller diameter and reducing the total number of beads.The β phase PVDF can be formed in a strict electrical situation (Bhang et al., 2017) (always over 10 kV), increasing the charging density assisted crystal phase changes raised the β phase percentage.

To demonstrate the effects of ZnO NPs on the ratio of the β phase in PVDF scaffolds, the applied voltage was chosen as the middle voltage (15 kV) to avoid an extremely high and low β phase PVDF ratio. Moreover, when the concentration of ZnO NPs increased from 0 to 2 mg/ml, the percentage of PVDF β phase increased by >10%. Compared to a previous study, Damarju et al. showed that by increasing the applied voltage from 15 kV to 25 kV, the β phase of PVDF improved to about 5% (Damaraju et al., 2013). It is believed that the increased charging density was able to affect the β phase ratio. Previous research showed that by adding conductive materials, one can increase the conductivity of the electrospun solution and increase the charging density of the electrospun solution during electrospinning (Zong et al., 2002; Lin et al., 2004). Owing that the β phase PVDF can be formed in a strict electrical situation (Bhang et al., 2017), increasing the charging density assisted crystal phase changes raised the β phase percentage, which matched previous studies.

The reason why piezoelectricity can help inhibit bacteria growth may be the following reason. When piezoexcited, the two sides of the scaffold were charged, then one side become cationic and the other side become anionic. Because almost all bacteria have negative charges on their surface due to their membrane proteins, acids and lipids, it is believed that the cationic scaffold surface could attract bacteria, then allow for their interaction with a high concentration ZnO NPs (because they are near to the scaffold) and be killed by ZnO NPs. Additionally, according to a previous study (Gottenbos et al., 2001), an anionic surface could slow down the initial adhesion rate of bacteria, but it would not inhibit bacteria growth after adhesion. Also, according to the piezoelectric coefficient of materials, it is possible to control which side is cationic or anionic by deforming the shape of the material. Thus, it is necessary to allow the anionic surface to attach to the bottom and allow the cationic surface to face the medium and bacteria (to get a better antimicrobial ability).Moreover, the results from piezoelectric excitation tests without piezoelectric scaffolds also showed that the stimulation method (dynamic stretching) can lead to silicone well plate bottom movement, inhibiting bacterial attachment to the surface, making it difficult to form a biofilm. This dynamic stimulation method can also accelerate ZnO NPs diffusion, which can help to inhibit bacterial growth.

Moreover, ZnO/PVDF scaffolds not only inhibit the growth of *E.coli* and *SA* bacteria, but also stimulate osteoblast proliferation under piezoexcitation. All of the characteristics of this (ZnO/PVDF) composite fiber would enhance the development of orthopedics in the future.
